# Food Compass Score vs FDA Healthy Labeling and Consumer Purchases: A Randomized Clinical Trial

**DOI:** 10.1001/jamanetworkopen.2025.46526

**Published:** 2025-12-05

**Authors:** Bingbing Fan, Katherine Fuller, Julia Reedy Sharib, Jennifer L. Pomeranz, Meng Wang, Lu Wang, Dariush Mozaffarian, Sean B. Cash

**Affiliations:** 1 Food is Medicine Institute, Friedman School of Nutrition Science & Policy, Tufts University, Boston, Massachusetts; 2 Department of Applied Economics, Oregon State University, Corvallis; 3 Department of Public Health Policy and Management, School of Global Public Health, New York University, New York

## Abstract

**Importance:**

The US Food and Drug Administration (FDA) has finalized a new front-of-package binary healthy label for packaged foods. Effects of this governmental label compared with a more holistic food rating score on consumer purchases are unknown.

**Objective:**

To evaluate the effects of the continuous Food Compass Score (FCS) and FDA healthy label in a real-choice randomized trial.

**Design, Setting, and Participants:**

This randomized clinical trial used real-choice experiments among adults recruited on-site at 6 locations of 3 supermarket chains in diverse neighborhoods across Massachusetts from July to November 2023.

**Intervention:**

Participants were randomized into 2 treatments: FCS (1-100) and FDA healthy (binary) labels. Real-choice experiments tested purchasing decisions comparing different snack foods with and without both label treatments, with these decisions leading to an actual final purchase. Each treatment group had 12 choice scenarios (6 labeled and 6 unlabeled) in which they could choose either 1 of 3 products or no product.

**Main Outcomes and Measures:**

Purchase choices (healthy product [defined as FCS≥70], unhealthy product, or no purchase) in multiple choice scenarios, recorded in-person by investigators, and willingness to pay for different features.

**Results:**

This study included 275 adults, with a median (IQR) age of 55 (38-66) years, and 175 (63.6%) were female. Compared with no label, both labels increased the purchase of healthier products (FCS: 11.2 [95% CI, 8.2 to 14.2] per 100 choices; *P* < .001; FDA healthy: 6.4 [95% CI, 3.4 to 9.4] per 100 choices; *P* < .001). Both labels also reduced the purchase of unhealthy products (FCS: −7.2 [95% CI, −9.9 to −4.6] per 100 choices; *P* < .001; FDA healthy: −6.3 [95% CI, −9.0 to −3.6] per 100 choices; *P* < .001). The FCS, but not FDA, label also reduced the occurrence of no purchases by 4.0 (95% CI, 1.8 to 6.1) per 100 choices (*P* < .001). Compared with the FDA healthy label, the FCS label increased healthy purchases by 1.75-fold (93 of 828 purchases [11.2%] vs 53 of 822 purchases [6.4%]).

**Conclusions and Relevance:**

In this randomized clinical trial, both labels increased healthy and decreased unhealthy product purchases, with larger effects as well as decreases in no-purchase decisions with the FCS label. These results can inform labeling decisions by policymakers, retailers, and manufacturers.

## Key Points

**Question:**

Does package labeling with a continuous food rating score or binary governmental healthy label promote healthier purchases?

**Findings:**

In this randomized clinical trial among 275 supermarket shoppers, both labels increased healthy and decreased unhealthy product purchases, but the food rating increased healthy purchases by 1.75-fold over the binary label and increased likelihood of selecting any product.

**Meaning:**

These findings suggest that a food rating score may be a preferred choice for front-of-package labeling by policymakers, food retailers, and manufacturers.

## Teaser

This randomized clinical trial compares the effects of a continuous food rating score label vs a US Food and Drug Administration (FDA) healthy label on promoting healthier food purchases by adults in the US.

## Introduction

Front-of-package (FOP) labeling aims to help shift consumer purchases of food and beverage products as well as incentivize manufacturer reformulations.^1,2,3,4^ In 2016, the US Food and Drug Administration (FDA) announced its intention to refine its official definition of healthy as a nutrient content claim for voluntary use by manufacturers on food packages and in marketing. In December 2024, the final rule was established, which included, for the first time in the US, an FDA Healthy FOP label.^5^ However, the potential effects of this label on consumer purchasing remain unknown. In addition, compared with such a dichotomized or binary system (healthy: yes or no), more holistic food rating systems have been developed that can provide a range of ratings of the healthfulness of food and beverage products.^6^

The Food Compass Score (FCS) is a food rating system that characterizes the overall healthfulness of foods and beverages,^7,8^ validated against healthy eating indices and disease outcomes in the US and other countries.^9,10,11^ FCS incorporates information on nutrients, food ingredients, processing, and additives to derive a final score ranging from 1 (least healthful) to 100 (most healthful), providing more discrimination than dichotomized labeling. While FCS could be an important tool to promote healthier food purchases, its effects on consumer behavior or its comparison with binary labeling systems are also unknown.

Indeed, the actual impact of food labeling on consumers’ actual purchases remains an active area of inquiry. Most studies evaluating purchases have been uncontrolled, pre- vs post-, or nonrandomized intervention population studies, whereas most randomized trials have been online experiments evaluating impacts on knowledge, awareness, attitudes, or theoretical purchases (intent) rather than actual purchases.^1,2,4,12,13,14,15,16,17,18,19,20,21^ A meaningful gap can exist between consumer intent, which is often aspirational, and actual purchasing behavior. Real-choice experiments, which evaluate actual purchases in controlled settings, can more closely approximate true consumer shopping behavior by mitigating many of the biases associated with hypothetical shopping experiments.^22^

To address these important research gaps, we evaluated the effects of FCS and FDA healthy labeling on consumer purchases in a randomized real-choice experiment conducted in grocery stores with participants recruited by intercepting shoppers. The findings of this study can help inform the potential selection and impact of various FOP labels for researchers, government, and industry.

## Methods

### Study Design, Setting, and Population

This randomized clinical trial was approved by the Tufts institutional review board and followed the Consolidated Standards of Reporting Trials (CONSORT) reporting guideline.^23^ We conducted a randomized real-choice experiment at 6 locations of 3 supermarket chains across central and eastern Massachusetts located in neighborhoods with varying socioeconomic characteristics, including Worcester, Chicopee, and multiple neighborhoods in Boston from July to November 2023. A total of 422 shoppers were recruited on-site at the supermarkets for this in-person trial, which had 3 treatments, including an FCS label, FDA healthy label, and generic healthy label, each compared with no label. Inclusion criteria were (1) age (≥18 years), (2) at least partly responsible for shopping for food for the household, and (3) not having major food allergies (to dairy, eggs, peanuts, tree nuts, wheat, soy, or gluten). Detailed information on the study design and participants is presented in eAppendix 1 in Supplement 1. In this analysis, we focus on the comparison of the FCS and FDA healthy labeling groups. Findings for the comparison of the FDA healthy and generic healthy label are being reported separately, aiming to explore the influence of trust, particularly in government entities, on consumers’ preferences for healthy food labels, as specified in the study hypotheses. All participants provided written informed consent. The study was pretested and refined with 83 individuals at a subset of the study sites and a local farmer’s market during the summer of 2022. Because it does not include measurements of a health outcome, this study was preregistered in the Open Science Foundation on May 2, 2023.^24^ The trial protocol and statistical analysis plan can be accessed in Supplement 2.

### Labeling Criteria

The FDA healthy label was defined according to the proposed rule released on September 28, 2022, based on products containing a minimum reasonable serving amount of fruits, vegetables, grains, fat-free or low-fat dairy, or recommended protein foods by the Dietary Guidelines for Americans, and not exceeding specific limits for sodium, added sugars, or saturated fat.^25^ All included products that were designated as FDA healthy remained qualified as FDA healthy under the final rule.^26^ The image for this label was derived from examples of the new label suggested by the FDA in a request for comment on agency data collection activities.^27^ The FCS was calculated for all study products as reported elsewhere based on attributes across 9 domains including nutrient ratios, vitamins, minerals, food-based ingredients, additives, processing, specific lipids, fiber and protein, and phytochemicals,^8^ each assessed per 100 kcal of food. The domain scores were summed and scaled to calculate a final FCS ranging from 1 (least healthful) to 100 (most healthful).

To determine healthful choices (whether or not they were labeled), we compared products defined as FDA healthy to those with an FCS of 70 or greater as the previously proposed reasonable cut point for foods or beverages to be encouraged.^7,8,9^ We used an FCS of 70 or greater to define healthier products, and in sensitivity analyses, we used FDA healthy. Among the 15 products (eTable 1 in Supplement 1), 9 were defined as FDA healthy, and 8 had an FCS of 70 or greater (Spearman *r* = 0.87; *P* < .001). The sole discordant product (organic dried mango) was defined as FDA healthy but had a modestly lower FCS score (FCS = 43). We did the analysis using either FCS or FDA healthy definition, and it did not change the results meaningfully.

### Real-Choice Experiments

The overall trial included 15 snack products, 8 of which were selected to be healthy (FCS ≥70) and 7 that were not. Participants were randomized using simple randomization in Qualtrics to 3 treatments: FCS (1-100), FDA healthy (binary), or generic healthy labels; results from the generic treatment are reported separately. Twelve choice scenarios were designed for each participant. The first 6 scenarios displayed no labels, which served as controls, and the latter 6 scenarios displayed the treatment labels (ie, the FCS or FDA healthy label). Before the labeling scenarios were presented, participants were provided with a brief description of each label’s origin and meaning (eAppendix 2 in Supplement 1). In each scenario, participants were presented with 3 of the snack products on an electronic tablet, with their prices, either with or without the labels (eFigure 1 in Supplement 1). Physical examples of each of the snack products were available for the participants to examine. In each scenario, participants could select either only 1 of the 3 products or none of the 3 products for purchase. Each participant was provided with $5 for participating in the study and was informed that, after completing their 12 choices, they must use a portion of these funds to purchase and take home one of their selected products (chosen randomly from among all the products they had selected in each choice scenario). Informing the participants of the binding purchase (real choice) makes the choice tasks more realistic than possible in hypothetical experiments and incentivizes participants to pay higher attention to the product attributes, providing greater external validity.^28,29^ Accordingly, these purchases took place at the end of data collection for each participant. The duration of the choice experiment and the survey for each shopper varied but typically ranged from 15 to 25 minutes.

### Outcomes and Other Measurements

For each choice scenario, the participant’s selection (any one product or none) was electronically recorded. Information on sociodemographics, shopping behavior, and attitudes was collected by standardized questionnaires. Sociodemographics included age, gender, race and ethnicity, income, education, household size, children at home, student status, nationality, and political affiliation. Race and ethnicity categories included Asian, Hispanic, non-Hispanic Black, non-Hispanic White, and other (defined as any race or ethnicity not otherwise specified); race and ethnicity were included to account for potential disparities. Other questions assessed participants’ knowledge around identifying healthy food options, shopping behavior, physical activity, diet, and presence of diet-related medical conditions. To assess the potential for differing responses to various labels based on feelings of trust, we also evaluated levels of generalized trust, interpersonal trust, institutional trust, and governmental trust using Likert scales. The detailed survey instrument is presented in the eMethods in Supplement 1.

### Statistical Analysis

Power analysis indicated that the target enrollment of 124 adults per treatment would provide 90% power with α = .05. To compare labeled with unlabeled scenarios within each treatment group, the McNemar test was used to calculate the *P* value for the choice difference, adjusted Wald intervals^30^ were calculated for the difference (95% CI) in purchases per 100 choices, and relative differences of FCS to FDA healthy were calculated for comparison. To account for the multinomial outcomes, within-person repeated measurements, and random effects of the product’s properties, multivariable mixed logit models were used to estimate the independent effects of each product’s labeling condition, price, and healthfulness (healthy or unhealthy) on consumer purchasing, with each variable specified as both fixed and random effects.^31^ We also explored the potential for interaction between labeling status and healthfulness. Subgroup analyses explored whether the effects of labeling on consumer purchasing were modified by participants’ education, household income, physical activity level, special diet, or trust levels. Statistical significance of these interaction analyses was Bonferroni corrected for multiple comparisons (α = .005). Finally, we evaluated the effects on purchases of healthy labels and product healthfulness in relation to price by assessing consumers’ willingness to pay, calculated as −β*_k_* divided by β*_price_*, where β*_price_* was the beta coefficient of price in the mixed logit models, and β*_k_* was the corresponding beta coefficient of labeling status, healthfulness, and their interactions. Willingness to pay quantifies the maximum dollar amount participants are willing to pay for products with that corresponding property. All analyses were implemented with R version 4.4.1 (R Project for Statistical Computing).

## Results

**Figure 1.**
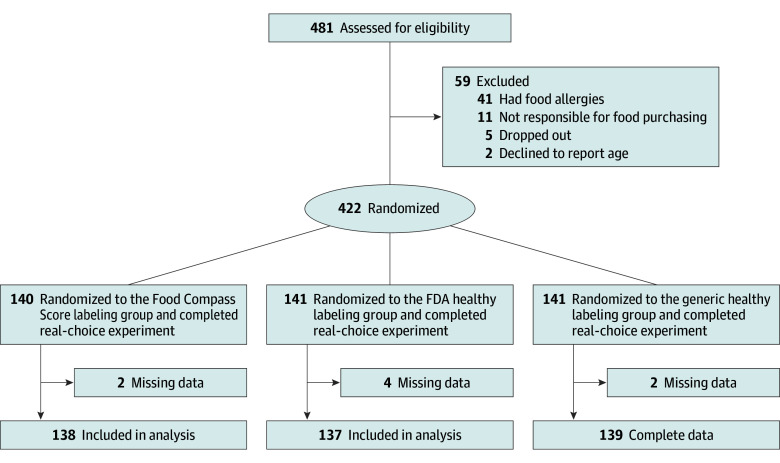
Flow Diagram In the present analysis, Food Compass Score and US Food and Drug Administration (FDA) healthy labeling groups were included. Findings for the comparison of the FDA healthy and generic healthy label are being reported separately.

As shown in Figure 1, 275 participants were included in the final analysis, with 138 randomized to FCS and 137 to FDA healthy. Baseline characteristics were balanced between the 2 randomized labeling groups (Table 1 and eTable 2 in Supplement 1). The median (IQR) age was 55 (38-66) years, and 179 (63.6%) were female. Among participants, 5 (1.8%) were Asian, 40 (14.5%) were Hispanic, 109 (32.4%) were non-Hispanic Black, 89 (32.4%) were non-Hispanic White, and 32 (11.6%) identified as other race or ethnicity. Education was comparably distributed between completion of high school, some college, or college graduation. Nearly two-thirds of participants (179 participants [65.1%]) had lower household income (<$40 000). Levels of trust varied according to the object of trust and were higher for generalized than for interpersonal, institutional, or governmental trust.

**Table 1.** Characteristics of Participants in the Real-Choice Experiment by Front-of-Package Labels

Variable	Food Compass Score (n = 138)	FDA healthy (n = 137)	Total (N = 275)
Age, median (IQR), y	55 (38-67)	55 (37-65)	55 (38-66)
Gender			
Male	48 (34.8)	44 (32.1)	92 (33.5)
Female	86 (62.3)	89 (65.0)	175 (63.6)
Nonbinary or other gender	4 (2.9)	4 (2.9)	8 (2.9)
Race and ethnicity			
Asian	5 (3.6)	0	5 (1.8)
Hispanic	23 (16.7)	17 (12.4)	40 (14.5)
Non-Hispanic Black	49 (35.5)	60 (43.8)	109 (39.6)
Non-Hispanic White	44 (31.9)	45 (32.8)	89 (32.4)
Other^a^	17 (12.3)	15 (10.9)	32 (11.6)
Education			
High school	47 (34.1)	53 (38.7)	100 (36.4)
College or technical school	42 (30.4)	43 (31.4)	85 (30.9)
Bachelor or higher	49 (35.5)	41 (29.9)	90 (32.7)
Annual household income, $			
<40 000	86 (62.3)	93 (67.9)	179 (65.1)
40 000-79 999	35 (25.4)	30 (21.9)	65 (23.6)
≥80 000	17 (12.3)	14 (10.2)	31 (11.3)
Physical activity			
Not active or somewhat active	77 (55.8)	70 (51.1)	147 (53.5)
Active	61 (44.2)	67 (48.9)	128 (46.5)
Special diet^b^	48 (34.8)	49 (35.8)	97 (35.3)
Generalized trust^c^			
Low or do not know	105 (76.1)	106 (77.4)	211 (76.7)
High	33 (23.9)	31 (22.6)	64 (23.3)
Interpersonal trust mean score^d^			
Low (<3)	29 (21.0)	31 (22.6)	60 (21.8)
Medium (3-5)	81 (58.7)	82 (59.9)	163 (59.3)
High (>5)	28 (20.3)	24 (17.5)	52 (18.9)
Institutional trust mean score^e^			
Low (<3)	34 (24.6)	33 (24.1)	67 (24.4)
Medium (3-5)	77 (55.8)	76 (55.5)	153 (55.6)
High (>5)	27 (19.6)	28 (20.4)	55 (20.0)
Trust in the government mean score^f^			
Low (<3)	62 (44.9)	52 (38.0)	114 (41.5)
Medium (3-5)	52 (37.7)	58 (42.3)	110 (40.0)
High (>5)	24 (17.4)	27 (19.7)	51 (18.5)

Abbreviation: FDA, Food and Drug administration.

^a^ Defined as any race or ethnicity not otherwise specified.

^b^ Special diet included vegan, vegetarian, flexitarian, paleo, pescetarian, religious-based, plant-based, and gluten-free.

^c^ Generalized trust was measured by asking participants, “Generally speaking, would you say that most people can be trusted or that you can’t be too careful in dealing with people?” The options were “Most people can be trusted” (defined as high generalized trust), “You need to be very careful in dealing with people” (defined as low generalized trust), and “I don’t know” (defined as do not know).

^d^ Interpersonal trust was measured by asking participants, “How do you generally feel towards other people?” There were 3 statements: “I generally trust other people,” “I feel that people are generally trustworthy,” and “I feel that people are generally reliable,” on a scale from 1 to 6, 1 being totally disagree and 6 being totally agree. Interpersonal trust score was calculated as the mean of the 3 scales.

^e^ Institutional trust was measured by asking participants, “How much trust do you have in the following groups regarding the production, selling, and regulation of food?” There were 4 statements: farmers or farmer groups, food manufacturers, retailers, and authorities. The scale used ranged from 1 to 7, where 1 was very little trust and 7 was very high level of trust. The Institutional trust score was calculated as the mean of the 4 scales.

^f^ Trust in the government was measured by asking participants, “To what extent do you agree or disagree with the following statements about authorities (departments of the government responsible for food regulations and laws such as USDA [US Department of Agriculture] or FDA)?” There were 9 statements regarding the work done by authorities regarding food regulation (options ranged from 1 [very little trust] to 7 [very high level of trust]). Governmental trust score was calculated as the mean of the 9 scales.

**Figure 2.**
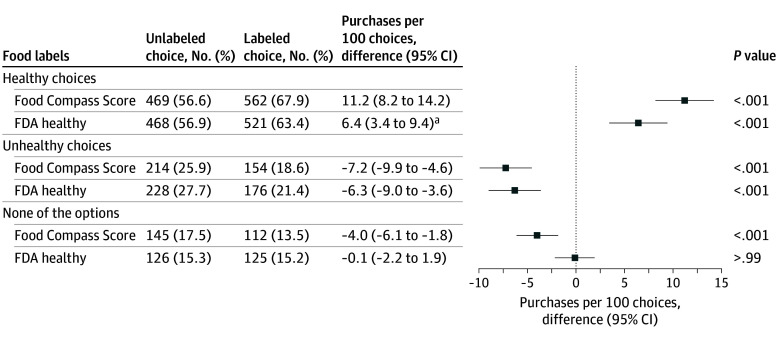
Effects of Front-of-Package Labels on Consumer Purchases Healthy choices were defined as a Food Compass Score of 70 or greater. The McNemar test was used to calculate the *P* value for difference (unlabeled vs labeled). Adjusted Wald intervals were calculated for difference of proportions (labeled − unlabeled). FDA indicates US Food and Drug Administration. ^a^*P* < .05 compared with Food Compass Score labeling.

Compared with no label, both labeling conditions increased consumers’ purchase of healthier products (Figure 2), consistent with our preregistered primary hypothesis. Healthier purchases increased by 11.2 (95% CI, 8.2 to 14.2) per 100 choices for FCS (*P* < .001) and 6.4 (95% CI, 3.4 to 9.4) per 100 choices for FDA healthy labels (*P* < .001). Each label also reduced the purchase of unhealthy products (FCS: −7.2 [95% CI, −9.9 to −4.6] per 100 choices; *P* < .001; FDA healthy: −6.3 [95% CI, −9.0 to −3.6] per 100 choices; *P* < .001). Only the FCS label reduced the rate of no purchases, resulting in 4.0 (95% CI, 1.8 to 6.1) more purchases per 100 choices (*P* < .001). Thus, for the FDA healthy label, increased selections of healthy products largely occurred in place of fewer selections of unhealthy products. In contrast, for FCS, increased selections of healthy products resulted from both fewer selections of unhealthy products and fewer no-purchase decisions. Compared with the FDA healthy label, the FCS label increased healthy purchases by 1.75-fold (93 of 828 purchases [11.2%] vs 53 of 822 purchases [6.4%]). In sensitivity analyses, the results were similar using FDA healthy to define healthy purchases (eFigure 2 in Supplement 1).

In multivariate models including each product’s labeling condition, price, and healthfulness, consumers were similarly more likely to select any labeled product for FCS labels (odds ratio [OR], 1.60; 95% CI, 1.09-2.36) and FDA healthy labels (OR, 1.58; 95% CI, 1.21-2.06) (Table 2). Product price was inversely associated with purchasing preference in both labeling conditions (FCS: OR, 0.77; 95% CI, 0.66-0.89; FDA healthy: OR, 0.80; 95% CI, 0.71-0.91). Accounting for price and labeling, healthful products were also more likely to be purchased in the FCS condition (OR, 2.33; 95% CI, 1.77-3.05) and in the FDA healthy condition (OR, 1.68; 95% CI, 1.32-2.14). Notably, for FCS labeling, which ranges from 1 to 100 rather than being binary, the effect of labeling on purchases varied by the healthfulness of products (ie, products having a higher numeric FCS were more likely to be selected). For the binary FDA label, there was no interaction between product healthfulness and labeling.

**Table 2.** Individual and Joint Effects of Front-of-Package Labels and Healthy Products on Consumers’ Purchases

Variables	Food Compass score		FDA healthy	
	Model 1, OR (95% CI)^a^	Model 2, OR (95% CI)^a^	Model 1, OR (95% CI)^a^	Model 2, OR (95% CI)^a^
Price, per dollar	0.77 (0.66-0.89)	0.77 (0.67-0.89)	0.80 (0.71-0.91)	0.80 (0.71-0.91)
Labeled^b^				
No	1 [Reference]	1 [Reference]	1 [Reference]	1 [Reference]
Yes	1.60 (1.09-2.36)	1.04 (0.68-1.60)	1.58 (1.21-2.06)	1.53 (0.96-2.42)
Healthy^c^				
No	1 [Reference]	1 [Reference]	1 [Reference]	1 [Reference]
Yes	2.33 (1.77-3.05)	1.66 (1.23-2.25)	1.68 (1.32-2.14)	1.67 (1.31-2.14)
Healthy × labeled^b^^,^^c^	NA	2.03 (1.53-2.68)	NA	1.04 (0.66-1.65)

Abbreviations: FDA, US Food and Drug Administration; NA, not applicable; OR odds ratio.

^a^ ORs greater than 1 indicated that consumers were more likely to purchase products with corresponding properties. ORs (95% CIs) were calculated by mixed logit models with label, price, and healthy included as fixed and random effects.

^b^ Labeled: if the product was labeled with corresponding healthy labels (Food Compass or FDA healthy label).

^c^ Defined as Food Compass score of 70 or greater.

In exploratory subgroup analyses, the effects of FCS labeling on purchase preferences were not significantly different by education, income, physical activity, following a special diet, or levels of various types of trust (eFigure 3 in Supplement 1). However, although not statistically significant, numerically larger central estimates were seen for labeling effects among those with lower vs higher education, with lower vs higher income, and with higher vs lower institutional and government trust. Likewise, no significant differences were seen among subgroups in the effects of FDA healthy labeling on purchase preferences (eFigure 4 in Supplement 1).

Regardless of labeling, consumers were willing to pay more for healthier products (Table 3). However, adjusted for healthfulness, consumers were not willing to pay significantly more for FDA healthy labeled products when they were healthier (willingness to pay, $2.13; *P* = .18). In comparison, consumers were willing to pay more for FCS labeled products when they were healthier, equivalent to a price premium of $2.90 (*P* = .02) more above and beyond the products being healthier alone.

**Table 3.** Effects of Front-of-Package Labels and Healthy Products on Consumers’ Willingness to Pay for Products

Variables	Food Compass Score		FDA healthy	
	WTP (SE), $^a^	*P *value	WTP (SE), $^a^	*P *value
Labeled products	0.17 (0.84)	.84	1.94 (1.16)	.09
Healthy products	1.97 (0.83)	.02	2.36 (0.90)	.009
Healthy and labeled products	2.73 (0.97)	.005	0.19 (1.08)	.86

Abbreviations: FDA, US Food and Drug Administration; WTP, willingness to pay.

^a^ WTP (SE) was calculated in US dollars by the corresponding coefficients divided by the coefficient of price calculated by mixed logit models (see model 2 in Table 2).

## Discussion

In this randomized clinical trial using a real-choice experiment, we evaluated the effects of 2 distinct FOP label options on consumer purchases. Several key findings were notable. First, we found that the food rating score (FCS) and FDA healthy label each significantly increased healthful purchases. Second, we saw that labeling with FCS increased consumer purchases of healthy products the most, by decreasing both purchases of unhealthy products and no-purchase decisions, while FDA healthy labeling had no effect on no-purchase decisions. Third, incorporating price, we found that consumers were willing to pay up to $2.90 more for a snack product that is both healthier and labeled with FCS. These findings inform potential labeling decisions by policymakers, food retailers, and manufacturers.

In comparison with binary systems, which may raise challenges to assigning absolute healthfulness (yes or no) to an individual food product in the context of complex diets,^6^ a numeric label can describe a range of healthfulness of food products. Consumers do not assess a food product’s healthfulness in isolation, but in relation to other existing information as well as their prior knowledge and expectations.^32,33,34^ While it could be hypothesized that binary labels might be less confusing and therefore more actionable for consumers, our findings indicate the opposite: that a greater discrimination of healthfulness results in larger behavior change. Because consumers tend to categorize foods as either healthy or unhealthy, they might spontaneously expect food products to be rated at the extremes of a nutritional scale.^14^ Thus, the numeric FCS labels had larger effects on promoting healthier purchases than binary FDA healthy labels. Studies on Nutri-Score^4,35^ and Health Star Rating^2^ labels, 2 graded FOP labels, reported similar advantages compared with binary FOP labels. In addition, while there have been concerns that food labeling may have smaller effects among socioeconomically marginalized groups,^36^ potentially increasing disparities, we identified no significant differences in any of the labeling effects by education or household income, and with numerically larger effects of FCS among consumers with lower education and lower household income, although these were not statistically significant.

A positive interaction between labeling and product healthfulness was only seen for FCS. According to expectancy-disconfirmation theory,^37^ when a product meets or exceeds a consumer’s expectations, their evaluations and satisfaction increase. We identified healthy products as more likely to be selected even without labeling, suggesting an awareness and preference for healthy items overall. While the binary FDA healthy label further increased healthy purchases, there was no interaction by the healthfulness of the product, suggesting that the FCS label might further increase consumers’ evaluation and satisfaction of healthier products and promote their purchases.

With the advent of on-shelf labeling and especially online grocery shopping, product labeling decisions are no longer solely the purview of food manufacturers. Food retailers have the option to add labeling, such as the on-shelf Guiding Stars tag^38,39^ or online diabetes friendly tags.^40^ However, given their business model, retailers would be hesitant to add any labeling that might decrease overall purchase decisions and sales. We found that binary FDA healthy label did not decrease overall purchasing decisions, and that the FCS actually increased overall purchasing decisions. These results support the need for larger implementation and evaluation studies of these labels in real-life settings. The growth of online shopping is particularly attractive for such efforts because retailers could randomize shoppers to different labeling conditions at scale, allowing rapid determination of effects.

The price of a product is a critical factor that influences consumer purchases.^41^ We found that consumers were willing to pay more for healthier products, whether they were labeled or not. Similar to a previous study focused on use of an FDA healthy label in yogurt product,^42^ we did not find significant effects of FDA healthy labeling on consumers’ willingness to pay. However, we found that consumers were willing to pay significantly more for healthy FCS-labeled products than for healthy products alone, supporting a business case for creating and marketing healthier FCS-labeled products for consumers. The effects of each label on purchases were not significantly different by levels of various types of trust, although our trial was not primarily powered for such subgroup analyses. Further studies are needed to investigate whether different types of consumer trust alter the effects of FOP labels.

Several prior studies have shown that FOP labels, including warning labels, interpretive traffic light labels, and food rating labels, could help consumers to identify^2,4,12,43,44,45,46,47^ and choose^2,4,12,35,47,48,49,50^ healthier foods, but most of these evaluated theoretical decisions rather than actual purchases. In addition, other studies have not found effects of FOP labels on consumers’ identification or choice of healthier foods.^51,52,53,54,55^ An online theoretical experiment of generic healthy labeling showed that the healthy label did not substantially improve consumers’ ability to identify healthier cereals.^55^ An in-person auction-based study on FDA healthy and taste labels revealed that the FDA healthy label, particularly when presented alone, tended to lower consumers’ willingness to pay.^42^ Our investigation builds upon and substantially expands these prior studies by testing both binary and numeric labeling systems in a randomized real-choice experiment conducted with shoppers in actual supermarket settings.

### Strengths and Limitations

Our study has several strengths. We evaluated consumer decisions that led to actual purchase decisions. The 12 choice scenarios were orthogonally designed, considering the combination of the products, price, and labeling. We recruited actual shoppers in retail settings who represented a diversity of race, ethnicity, education, and income backgrounds. We assessed the independent and joint effects of labeling, product healthfulness, and price, as well as willingness to pay. The large sample size and multiple scenarios provided ample statistical power to detect differences.

This study also has limitations. The labels were not placed directly on the product packages, and thus the findings may be most directly applicable to on-shelf or online labeling, although differential effects of on-package labels should not be assumed. Our experiment utilized novel stimuli (ie, neither the FCS score nor proposed FDA healthy label were familiar to our participants), and therefore our study cannot predict how consumers might respond to scores or labels over time. Our trial focused on common snack foods, and similar investigations for other food categories should be considered. The real-choice experiments were conducted in 3 supermarket chains in Massachusetts, and while the neighborhoods and socioeconomic characteristics varied, the findings could differ in other US or non-US regions.

## Conclusions

In this randomized clinical trial, labeling with the continuous FCS and FDA healthy increased consumer purchases of healthy products and decreased purchases of unhealthy products, with larger effects as well as decreases in no-purchase decisions with the FCS label. These results can inform labeling decisions by policymakers, retailers, and manufacturers.

## Notes

Supplement 1. eAppendix 1. Study Design and Participants eTable 1. Food Compass Score and Qualification of FDA Healthy of Snack Products in the Real-Choice Experiment eAppendix 2. Food Labels eFigure 1. Example Products With Renderings of (a) Unlabeled, (b) FCS-Labeled, and (c) FDA Healthy-Labeled Snacks eMethods. eTable 2. Characteristics of Participants by Front-of-Package Labels eFigure 2. Effects of FOP Labels on Consumer Purchases eFigure 3. Effects of FCS Labeling on Consumer Purchases by Subgroups eFigure 4. Effects of FDA Healthy Labeling on Consumer Purchases by Subgroups

Supplement 2. Trial Protocol and Statistical Analysis Plan

Supplement 3. Data Sharing Statement
