# Supplementary material for: Food Compass Score vs FDA Healthy Labeling and Consumer Purchases: A Randomized Clinical Trial
Source: JAMA Netw Open. 2025 Dec 5;8(12):e2546526. doi: 10.1001/jamanetworkopen.2025.46526 (PMC12681036; doi:10.1001/jamanetworkopen.2025.46526)
Supplement: Supplement 1. — eAppendix 1. Study Design and Participants eTable 1. Food Compass Score and Qualification of FDA Healthy of Snack Products in the Real-Choice Experiment eAppendix 2. Food Labels eFigure 1. Example Products With Renderings of (a) Unlabeled, (b) FCS-Labeled, and (c) FDA Healthy-Labeled Snacks eMethods. eTable 2. Characteristics of Participants by Front-of-Package Labels eFigure 2. Effects of FOP Labels on Consumer Purchases eFigure 3. Effects of FCS Labeling on Consumer Purchases by Subgroups eFigure 4. Effects of FDA Healthy Labeling on Consumer Purchases by Subgroups [file jamanetwopen-e2546526-s001.pdf]

Supplemental Online Content

Fan B, Fuller K, Sharib JR, et al. Effects of Food Compass Score vs FDA healthy labeling on consumer purchases: a randomized clinical trial. *JAMA Netw. Open*. 2025;8(12):e2546526. doi:10.1001/jamanetworkopen.2025.46526

eAppendix 1. Study Design and Participants

eTable 1. Food Compass Score and Qualification of FDA Healthy of Snack Products in the Real-Choice Experiment

eAppendix 2. Food Labels

eFigure 1. Example Products With Renderings of (a) Unlabeled, (b) FCS-Labeled, and (c) FDA Healthy-Labeled Snacks

eMethods.

eTable 2. Characteristics of Participants by Front-of-Package Labels

eFigure 2. Effects of FOP Labels on Consumer Purchases

eFigure 3. Effects of FCS Labeling on Consumer Purchases by Subgroups

eFigure 4. Effects of FDA Healthy Labeling on Consumer Purchases by Subgroups

This supplemental material has been provided by the authors to give readers additional information about their work.

© 2025 Fan B et al. *JAMA Network Open*.

eAppendix 1. Study Design and Participants

We conducted a randomized real-choice experiment at 6 locations of three supermarket chains across central and eastern Massachusetts located in neighborhoods with varying socioeconomic characteristics, including Worcester, Chicopee, and multiple neighborhoods in Boston from July to November 2023. The retailers were chosen based on existing connections and local ownership, as obtaining permission to recruit actual shoppers as participants into in situ experiments was an important logistical element to conducting this study. Specific store locations were based on recommendations from the retailers but reflected the study team's desires to recruit in settings representing a variety of socio-economic contexts. The experiments were always conducted on weekdays due to both scheduling logistics and to minimize the burden on store staff at peak weekend times (at the request of our retail partners). Time of day and day of the week was otherwise varied. Participants were approached by researchers holding a flyer explaining the study who invited participants to come sit at the study table to receive additional information. Participants were approached throughout the day (i.e., not on a set schedule). Research days were usually >5 hours in length. Inclusion criteria were (1) age $\geqslant$ 18 years, (2) at least partly responsible for shopping for food for the household, and (3) not having major food allergies (to dairy, eggs, peanuts, tree nuts, wheat, soy, or gluten). Participants were approached throughout the day (i.e., not on a set schedule). Research days were usually >5 hours in length. Because the research tables were set up in the interior of the store, choice experiments were almost always conducted before participants purchased their own groceries. Store customers were approached by researchers and were invited to participate. Shoppers who declined to participate were not pursued further. Researchers continued to collect responses from shoppers until the full study sample was collected. 417 shoppers were recruited in the final experiment, comprising 139

© 2025 Fan B et al. *JAMA Network Open*.

participants in the Food Compass Score group, 140 in the FDA healthy group, and 138 in the generic healthy group. In this analysis, we focus on the comparison of the FCS and FDA healthy labeling groups, comprising 275 participants after excluding 2 individuals with missing data from the 12 choice scenarios.

© 2025 Fan B et al. *JAMA Network Open*.

eTable 1. Food Compass Score and qualification of FDA healthy of snack products in the real-choice experiment

Snack products	FCS	FDA healthy
Bare Naked Crunchy Pineapple Chips, 1.6 oz	74	Yes
Mott's Red Apple Slices, 2 oz	94	Yes
Oh! Nuts Roasted Salted Mixed Nuts Snack Pack, 1.5 oz	95	Yes
KIND Dark Chocolate Nuts & Sea Salt Bar, 1.4 oz	71	Yes
Biena Chickpea Snacks, Sea Salt, 1.2 oz	86	Yes
Made In Nature Organic Dried Mango Snack Packs, 1 oz	43	Yes
Seapoint Farms Dry Roasted Edamame – Sea Salt, 1.58 oz	100	Yes
Bolthouse Farms/Store Brand Carrots Mini Peeled Snack Pack, 2.25 oz	100	Yes
Harvest Snaps Green Pea Snack Crisps Lightly Salted, 1 oz	91	Yes
Nature's Bakery Whole Wheat Fig Bars - Original Fig, 2 oz	13	No
Cascadian Farms Organic Dark Chocolate Chip Chewy Granola Bars, 1.2 oz	36	No
Annie's Organic Cookie Bites Chocolate Chip Pack – 1.05 oz	13	No
Whisps Parmesan Cheese Crisps, 0.63 oz	28	No
Kettle Brand Kettle Sea Salt Potato Chips, 1.5 oz	55	No
Cheez-It Original Baked Snack Crackers, 1 oz	11	No

FCS indicates Food Compass Score; FDA, Food and Drug Administration.

© 2025 Fan B et al. *JAMA Network Open*.

eAppendix 2. Food Labels

Food Compass Score label

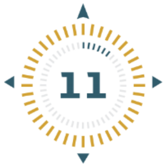

This label represents the Food Compass Score, a system designed by experts that evaluates the overall healthfulness of foods across nine areas, for example, including nutrient ratios, vitamins, minerals, food ingredients, additives, processing, and fiber and protein. Each food product is scored between 1 and 100, with 100 being the most healthful.

FDA healthy label

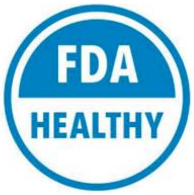

This label represents foods that contain a meaningful serving of one or more food groups recommended by the Dietary Guidelines for Americans, like fruits, vegetables, whole grains, nuts, or dairy; and also have low amounts of added sugar, sodium, and saturated fat. This label is created and endorsed by the Food and Drug Administration (FDA), an agency of the United States federal government.

© 2025 Fan B et al. *JAMA Network Open*.

Generic healthy label

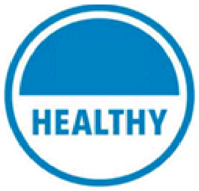

This label represents foods that contain a meaningful serving of one or more food groups recommended by the Dietary Guidelines for Americans, like fruits, vegetables, whole grains, nuts, or dairy; and also have low amounts of added sugar, sodium, and saturated fat.

© 2025 Fan B et al. *JAMA Network Open*.

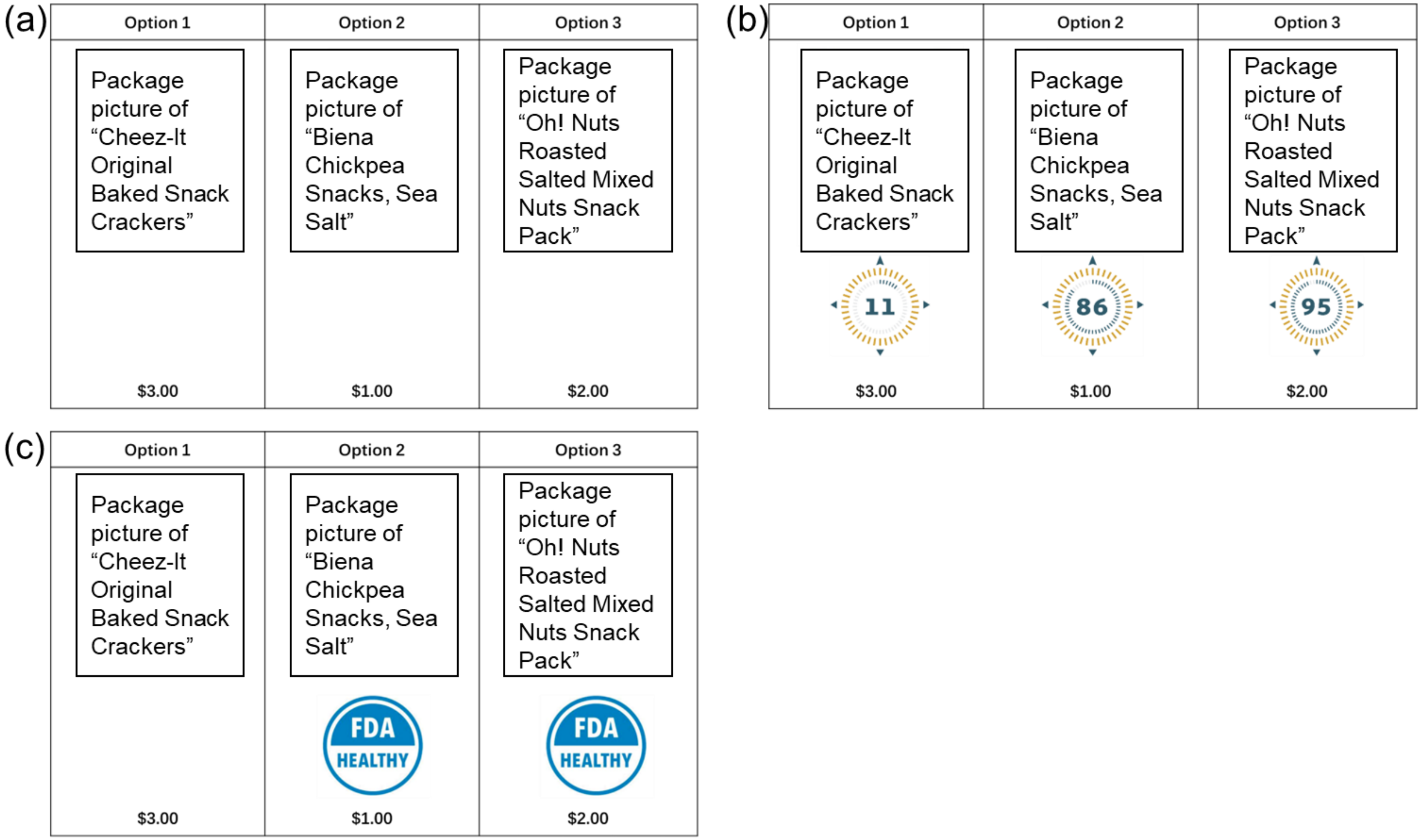

eFigure 1. Example Products With Renderings of (a) Unlabeled, (b) FCS-Labeled, and (c) FDA Healthy-Labeled Snacks. FCS, Food Compass Score; FDA, Food and Drug Administration.

© 2025 Fan B et al. *JAMA Network Open*.

eMethods

Information on sociodemographics, shopping behavior and attitudes, trust levels (generalized trust, interpersonal trust, institutional trust, and trust in the government) were collected by standardized questionnaires.

Subjective knowledge was measured by asking participants, “How knowledgeable are you when it comes to identifying healthy food options when grocery shopping?” (on a scale from 1-4, 1 being “not knowledgeable”, 4 being “very knowledgeable”).

Attention was measured by asking participants, “How often do you read food packaging labels before buying a product?” (on a scale from 1-3, 1 being “Never” and 3 being “always”)

“Healthy” shopping was measured by asking participants, “How often do you look for healthy options when food shopping?” (on a scale from 1-3, 1 being “Never” and 3 being “always”).

Difficulties to healthy shopping was measured by asking participants, “How difficult is it to identify healthy food options when food shopping?” (Easy or difficult are the only options).

Healthy purchase was measured by asking participants, “How often do you purchase food that is labeled as healthy?” (Never, monthly, weekly, and daily).

© 2025 Fan B et al. *JAMA Network Open*.

Physical activity level was measured by asking participants, “What would best describe your physical activity level” (not active, somewhat active, highly active, or extremely active).

Generalized trust was measured by the question, “Generally speaking, would you say that most people can be trusted or that you need to be very careful in dealing with people?” Participants can respond, “Most people can't be trusted”, “You need to be very careful in dealing with people”, or “I don’t know”.

Personal trust was measured by asking participants how much they agreed on a scale from 1 to 7 (where 1 is “strongly disagree” and 7 is “strongly agree”) to statements related to trust, trustworthiness, and reliability in others.

Institutional trust was measured by asking participants, “How much trust do you have in the following groups regarding the production, selling, and regulation of food?” There are 4 statements: Farmers or Farmer groups - People who produce plants and/or animals for human consumption; Food manufacturers - Companies that prepare, preserve, and pack food (e.g: Nestle, PepsiCo); Retailers - Companies that sell goods to the public in relatively small quantities (e.g: Walmart, Target); Authorities - Departments of the government responsible for food regulations and laws (e.g. USDA). The scale we use is from 1 to 7, where 1 is “Very little trust” and 7 is “Very high level of trust”.

Trust in the government was measured by asking participants, "To what extent do you agree or disagree with the following statements about authorities (departments of the government

© 2025 Fan B et al. *JAMA Network Open*.

responsible for food regulations and laws such as USDA or FDA)?" There are 9 statements regarding the work done by authorities regarding food regulation (options are 1-very little trust to 7-very high level of trust).

© 2025 Fan B et al. *JAMA Network Open*.

eTable 2. Characteristics of Participants by Front-of-Package Labels

Variable	Food compass	FDA healthy	*P*
**Sample size**	138	137	
**Political affiliation, n (%)**			0.169
Republican	5 (3.6)	13 (9.5)	
Democrat	57 (41.3)	60 (43.8)	
Independent	42 (30.4)	32 (23.4)	
Other	34 (24.6)	32 (23.4)	
**Household size, n (%)**			0.135
1	42 (30.4)	51 (37.2)	
2	31 (22.5)	26 (19.0)	
3	28 (20.3)	26 (19.0)	
4	13 (9.4)	16 (11.7)	
≥5	24 (17.4)	18 (13.1)	
**Children at home, n (%)**	33 (23.9)	25 (18.2)	0.316
**Diet-related condition, n (%)**			0.457
Self	39 (28.3)	30 (21.9)	
Member	22 (15.9)	22 (16.1)	
No	77 (55.8)	85 (62.0)	
**Subjective knowledge, n (%)**			0.467
Not/Somewhat	37 (26.8)	46 (33.6)	
Knowledgeable	59 (42.8)	52 (38.0)	
Very knowledgeable	42 (30.4)	39 (28.5)	
**Attention, n (%)**			0.162
Not/Somewhat	69 (50.0)	81 (59.1)	
Never	69 (50.0)	56 (40.9)	
**Healthy shopping, n (%)**			0.469

© 2025 Fan B et al. *JAMA Network Open*.

Not/Somewhat	62 (44.9)	70 (51.1)	
Always	76 (55.1)	67 (48.9)	
**Difficulty in healthy shopping, n (%)**			0.999
Not/Somewhat	98 (71.0)	97 (70.8)	
Always	40 (29.0)	40 (29.2)	
**Healthy purchase, n (%)**			0.019
Never	15 (10.9)	8 (5.8)	
Monthly	28 (20.3)	42 (30.7)	
Weekly	71 (51.4)	76 (55.5)	
Daily	24 (17.4)	11 (8.0)	

FDA indicates Food and Drug Administration.

© 2025 Fan B et al. *JAMA Network Open*.

Food Labels	Unlabeled, choice (%)	Labeled, choice (%)	Difference (95% CI) in purchases per 100 choices	*P*
**Healthy choices**				
Food Compass Score	496 (59.9)	578 (69.8)	9.9 (6.9, 12.8)	<0.001
FDA healthy	498 (60.6)	558 (67.9)	7.3 (4.3, 10.2)	<0.001
**Unhealthy choices**				
Food Compass Score	187 (22.6)	138 (16.7)	-5.9 (-8.5, -3.3)	<0.001
FDA healthy	198 (24.1)	139 (16.9)	-7.2 (-9.7, -4.6)	<0.001
**None of the options**				
Food Compass Score	145 (17.5)	112 (13.5)	-4.0 (-6.1, -1.8)	<0.001
FDA healthy	126 (15.3)	125 (15.2)	-0.1 (-2.2, 1.9)	0.999

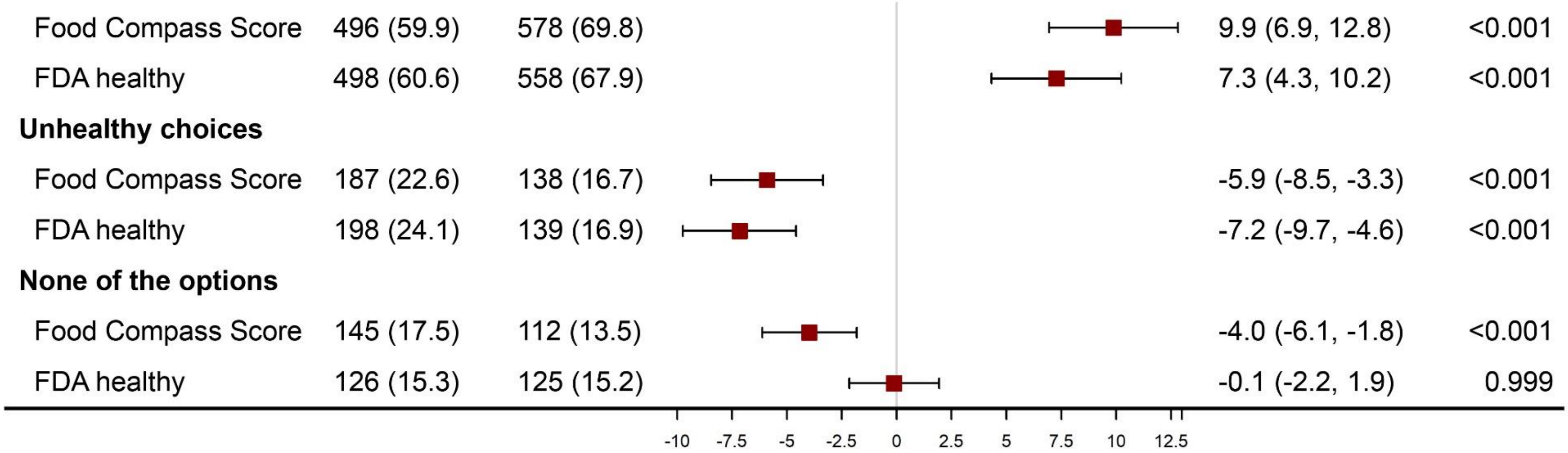

eFigure 2. Effects of FOP Labels on Consumer Purchases

CI indicates confidence interval; FDA, Food and Drug Administration; FOP, front-of-package.

Healthy choices were defined by FDA healthy criteria.

McNemar's test was used to calculate the *P* value for difference (unlabeled vs. labeled). Adjusted Wald intervals were calculated for the difference of proportions (labeled minus unlabeled).

© 2025 Fan B et al. *JAMA Network Open*.

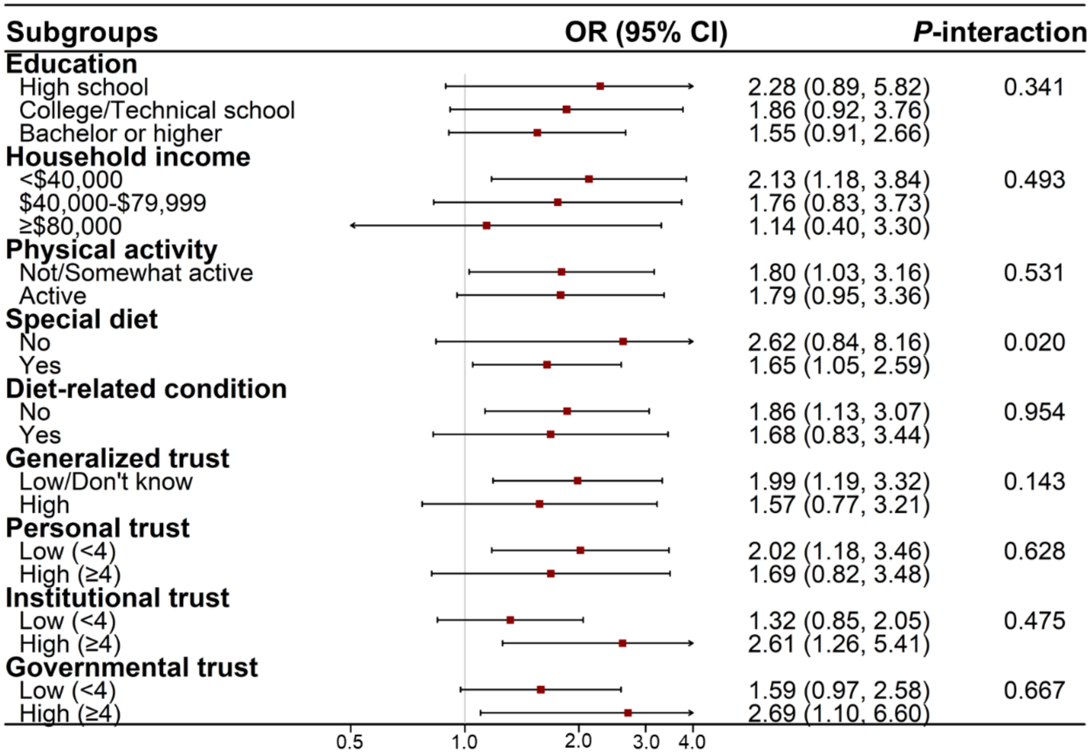

eFigure 3. Effects of FCS Labeling on Consumer Purchases by Subgroups

CI indicates confidence interval; FCS, food compass score; OR, odds ratio. ORs (95% CIs) were calculated by mixed logit models adjusting for price and healthy (see model 1 in **Table 2**). ORs > 1 indicated consumers were more likely to purchase products labeled by FCS. *P* for interaction assessed the overall interaction between FCS labels and each potential effect modifier. High personal, institutional, and governmental trust was defined as the corresponding trust score ≥ 4. High generalized trust was defined as participants answering “Most people can be trusted” in the questionnaire.

© 2025 Fan B et al. *JAMA Network Open*.

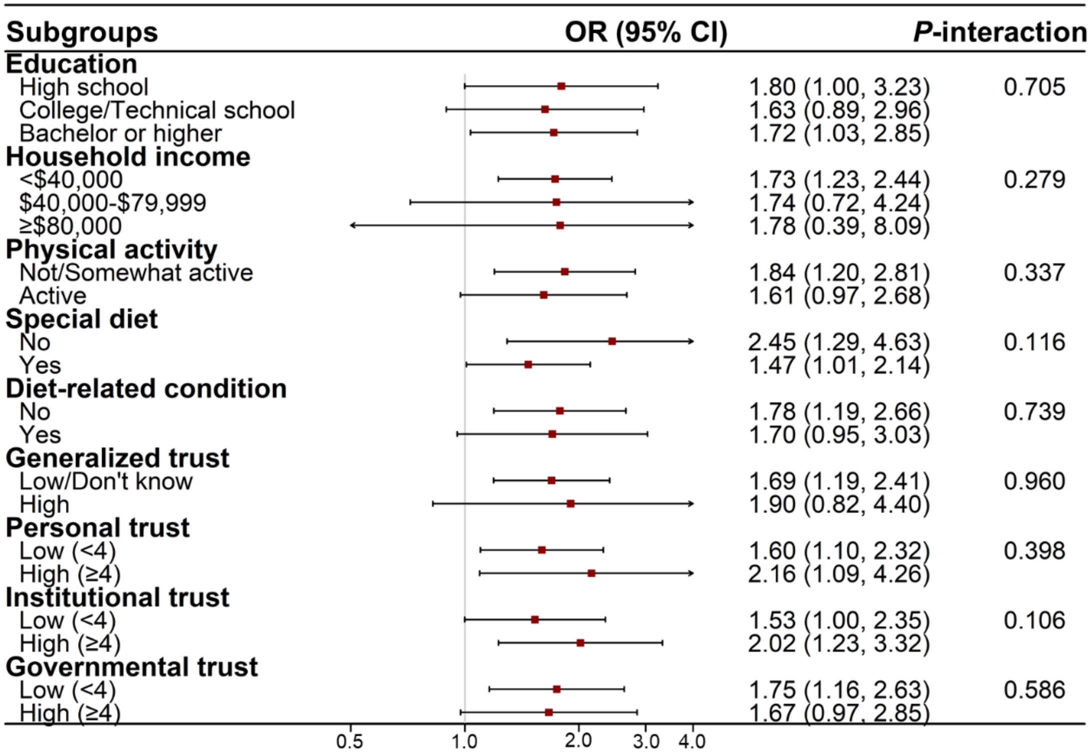

eFigure 4. Effects of FDA Healthy Labeling on Consumer Purchases by Subgroups

CI indicates confidence interval, FDA, Food and Drug Administration; OR, odds ratio. ORs (95% CIs) were calculated by mixed logit models adjusting for price and healthy (see model 1 in **Table 2**). ORs > 1 indicated consumers were more likely to purchase products labeled by FCS. *P* for interaction assessed overall interaction between FCS labels and each potential effect modifier. High personal, institutional, and governmental trust was defined as the corresponding trust score ≥ 4. High generalized trust was defined as participants answering “Most people can be trusted” in the questionnaire.

© 2025 Fan B et al. *JAMA Network Open*.
