# Supplementary material for: Food Compass Score vs FDA Healthy Labeling and Consumer Purchases: A Randomized Clinical Trial
Source: JAMA Netw Open. 2025 Dec 5;8(12):e2546526. doi: 10.1001/jamanetworkopen.2025.46526 (PMC12681036; doi:10.1001/jamanetworkopen.2025.46526)
Supplement: Supplement 2. — Trial Protocol and Statistical Analysis Plan [file jamanetwopen-e2546526-s002.pdf]

PROTOCOL TITLE:

Consumer perceptions of the FDA Healthy Label
[Phase 1-Pre-testing & Phase 2 – Final Collection]

PRINCIPAL INVESTIGATOR (PI):

Name: Sean Cash
Department: Friedman School of Nutrition Science and Policy
Are you a student? Yes ☐ No ☒
If Yes, you must designate a Faculty Advisor below.

FACULTY ADVISOR (required for Student PIs):

Name: N/A
Department: N/A

VERSION NUMBER/DATE

V1; Feb 6, 2023

1.0 Purpose of the study:

This study aims to develop and refine a survey instrument that evaluates consumer perception of a healthy label and the willingness to pay for it. The overall objectives of the project are to:

1. Understand and describe behaviorally segmented elasticity measures and estimates of the response to variations in nutrition-related food labeling.
2. Evaluate the impact of trust in various food-related institutions on food choice.
3. Evaluate the impact of a "healthy" food label, both with and without FDA endorsement, on snack food choices.
4. Identify nutrition-related food label components best suited for policy and regulatory updates aimed at improving the healthfulness of snack food choices.

This project is divided into two Phases. The primary objective of Phase 1 of this project is to pre-test the survey tool to refine and improve the presented content. Data collected from Phase 1 will not be retained or used for further analysis beyond the improvement of the survey for Phase 2. Phase 2 will proceed only after protocol modifications are submitted to incorporate feedback from the pretesting done in Phase 1. The focus of this application is for approval for Phase 1.

<u>Phase 2 – Full Collection</u>

Phase 2 includes the edits to the instrument after the feedback received from Phase 1 – Pretesting. The focus of this application is for approval for Phase 2.

2.0 Background / Literature Review / Rationale for the study:

The U.S. Food and Drug Administration (FDA) announced in 2016 that it would begin redefining "healthy" food labels, in part spurred by a dispute with KIND Snacks about its use of the term on KIND Bar packaging.[1] The lack of an updated regulatory definition of the term has left food companies like KIND vulnerable to class action[2] and consumers vulnerable to confusing or misleading food labels. The FDA, however, has made no regulatory decisions since the initial 2016 request for information. In the interim, the FDA has held a public meeting on the redefinition, updated its Nutrition Innovation Strategy to include defining the term, and got approval from the Office of Management and Budget (OMB) to create a focus group review of a "healthy" icon on food packages.[3,4] Most recently, OMB approved another proposal from the FDA to redefine the term and produce additional research on developing a "healthy" front-of-package (FOP) icon.[5] However, no research evaluating the impact of a "healthy" label, particularly one endorsed by the FDA, currently exists in a U.S. population.

The influence of an FDA-endorsed "healthy" label on food choice is also presumed to interact with the level of trust participants have in food-related institutions like the FDA. Survey questions of attitude-based trust questions (e.g., "generally speaking, would you say that most people can be trusted or that you can't be too careful in dealing with people?") have shown varying relationships with choice.

The lack of trust in the food value chain is a significant problem analyzed in the literature (Hobbs and Goddard, 2015; Sapp et al., 2009; Kaiser and Algers, 2017). With increasing claims about product credence characteristics, the lack of trust most likely decreases consumer confidence in their ability to make informed food choices (De Jonge et al. 2007). Consumers need to trust that manufacturers and farmers do what they say they do and that the government enforces regulations regarding misleading claims. Research has examined consumer trust levels among farmers, retailers, manufacturers, and the government. For instance, Macready et al. (2020) found that consumers in five European countries had different opinions about which institutions to trust. Spanish consumers have a higher level of trust except for trust in the government. Meanwhile, French consumers have lower levels of trust, except trust in the government.

Previous literature has shown that real choice experiments (RCE) have a high level of external validity (Chang et al., 2009), and it reduces hypothetical bias (Lusk, J. and Schroeder, T.C., 2004). In hypothetical settings, individuals have little incentive to put cognitive effort into their decision. RCEs have been used before to study food labels for Organic products (Chen et al. 2019) and traceability (Wu et al. 2015). We are closing the literature gap by adding a novel label and comparing it with other labels.

We are using Real Choice Experiments, which are more realistic than hypothetical experiments and have been demonstrated to lead to higher external validity. Real choice experiments require real economic incentives. This strategy involves informing participants that one of the choice scenarios will be selected at random and will be binding in terms of purchasing. Hence, participants have to buy the chosen products in the binding scenario and pay the corresponding price. To implement an RCE, the product to be valued needs to have quality attributes that can be easily varied and are widely available on the market (Ballco and Gracia, 2020).

3.0 Participant Population:

The pre-testing participants will be adults 18 years or older responsible for shopping for their household. Subjects will be recruited from the Greater Boston area. Participants will be English-speaking adults recruited at the specific locations listed below. No demographic quotas will be used to determine the target study sample demographics.

Phase 2 – Full Collection

The participant population is the same as pre-testing participants.

4.0 Special Populations:

N/A

- ☐ Children
- ☐ Fetuses/Neonates
- ☐ Prisoners
- ☐ Members of the military
- ☐ Non-English speakers

- [ ] Those unable to read (illiterate)
- [ ] Employees of the researcher
- [ ] Students of the researcher
- [ ] Adults lacking capacity to consent and/or adults with diminished capacity to consent, including, but not limited to, those with acute medical conditions, psychiatric disorders, neurologic disorders, developmental disorders, and behavioral disorders
- [ ] Disadvantaged in the distribution of social goods and services such as income, housing, or healthcare
- [ ] Fear of negative consequences for not participating in the research (e.g., institutionalization, deportation, disclosure of stigmatizing behavior)
- [ ] Approached for participation in research during a stressful situation such as emergency room setting, childbirth (labor), etc.

5.0 Research Locations and Sample Size:

5.1 Research Locations

During pretesting, participants will be recruited from a variety of retail food establishments in the Boston area, including farmers' markets, grocery stores, and specialty food stores. The research will be conducted at a table located within the establishments' retail space. The first phase will include the Medford Farmer's Market, Stop & Shop and Daily Table.

<u>Phase 2 – Full Collection</u>

During full collection, participants will be recruited from a variety of retail food establishments in the Boston area, including farmers' markets, grocery stores, and specialty food stores. The research will be conducted at a table located within the establishments' retail space. The first phase will include Stop & Shop, Daily Table and the Big Y.

5.2 Sample Size

The expected sample size for pre-testing our survey instrument in Phase 1 is up to 100 participants; we expect to reach saturation on feedback and error detection with 50-100 participants.

<u>Phase 2 – Full Collection</u>

The expected sample size for full collection is up to 400 participants; we expect to reach saturation on feedback and error detection with 350 - 400 participants.

6.0 Procedures Involved:

<u>Design</u>

Qualtrics is used to host a survey to evaluate consumers' perceptions and willingness to pay for a healthy label consisting of a Real Choice Experiment with additional sociodemographic and attitudinal questions. Question types include single choice, multiple choice, Likert scale, and open-answer questions.

<u>Pre-Testing</u>

The first phase of this study involves pre-testing the Qualtrics survey in the summer of 2022. A table will be set up for willing market shoppers to stop and take the survey on iPads provided by Tufts. At the end of the survey, they will be asked for feedback about the survey (see the attached semi-structured interview script). The survey is expected to take 20-25 minutes to complete.

As part of the choice experiment section, participants will be presented with choices between two to three snack food products and accompanying renderings of food labels for each product. Participants select only one product on each choice scenario (they will be exposed to 9 choice scenarios). Alternatively, if participants don't want to choose any of the snack products, they can opt out by choosing the "no purchase" option. After they finish making their nine choices, they will randomly pick a number to select one of the nine choice scenarios as the binding choice scenario. Participants will only pay for the (one) snack product they chose in the binding choice scenario. If the participant selected the "no purchase" option in the selected binding choice scenario, they won't receive a product and won't have to pay anything. The research team will have the snack products readily available so participants can have their product as soon as they pay for it.

Since the Real Choice Experiment mandates that the selection is binding and that the subject will pay for their chosen product, the research team will give participants $5 cash at the beginning of the study after they signed the consent form and answer the screening questions. The screening questions are if participants are 18 years or older and if they are responsible for shopping for food for the household.

<u>Phase 2 – Full Collection</u>

The second phase of this study involves full collection of the experiment using our survey on Qualtrics in the Winter / Spring of 2023. A table will be set up for willing market shoppers to stop and take the survey on iPads provided by Tufts. The survey is expected to take 15-20 minutes to complete.

As part of the choice experiment section, participants will be presented with choices between two to three snack food products and accompanying renderings of food labels for each product. Participants select only one product on each choice scenario (they will be exposed to 8 choice scenarios). Alternatively, if participants don't want to choose any of the snack products, they can opt out by choosing the "no purchase" option. After they finish making their nine choices, they will randomly pick a number to select one of the nine choice scenarios as the binding choice scenario. Participants will only pay for the (one) snack product they chose in the binding choice scenario. If the participant selected the "no purchase" option in the selected binding choice scenario, they won't receive a product and won't have to pay anything. The research team will have the snack products readily available so participants can have their product as soon as they pay for it.

Since the Real Choice Experiment mandates that the selection is binding and that the subject will pay for their chosen product, the research team will give participants $5 cash at the beginning of the study after they signed the consent form and answer the screening questions. The screening questions are if participants are 18 years or older and if they are responsible for shopping for food for the household.

6.1 Additional Safeguards for Special Populations:

NA

7.0 Investigational Medical Devices:

NA

8.0 Incomplete Disclosure or Deception:

NA

9.0 Recruitment Methods:

For pre-testing, there will be a table at the establishments where any shopper 18 years and responsible for food shopping for their household will be invited to complete the survey. Participants will be recruited while they patronize the establishments by inviting them to sit with a research team member at the study table. Prospective subjects will be informed of the compensation provided for participation. Please see the attached recruitment scripts and materials.

Phase 2 – Full Collection

The recruitment method is the same as in pretesting.

10.0 Consent Process:

At least one member of the research team will be present during survey pre-testing. Only adults aged 18 years and responsible for food shopping for their household are allowed to participate. The participant will be given the paper Consent Form, which includes details on this study's aims, how the collected anonymized data will be used, and contact information in case of any questions. The participant is asked if they consent to partake in the survey and will sign their name when providing consent. (See the attached Consent form).

Phase 2 – Full Collection

The consent process is the same as in pretesting.

11.0 Compensation:

Participants will be provided with a total value of up to $15.00. Up to $5.00 will be used to purchase study products at the study site from one of the research team members. Participants will keep the change for this $5.00. If the snack product is less than $5, participants will be able to keep the change from the purchase. If for the selected binding choice scenario, the participant selected “no purchase”, the participant will be able to keep the $5. An additional $10.00 will be given to participants in the form of gift cards / vouchers from the Medford Farmer’s Market, Stop & Shop, or Daily Table depending on where pretesting takes place. Vouchers / gift cards will be handed to the participant by a research team member once the survey has been completed.

Participants will be made aware of the compensation amount before beginning the survey. After completing the survey, they will be provided the chosen study product and

remaining monetary compensation. Participants will only receive compensation if they complete the experimental portion of the survey.

Phase 2 – Full Collection

Participants will be provided with a total value of up to $15.00. Up to $5.00 will be used to purchase study products at the study site from one of the research team members. Participants will keep the change for this $5.00. If the snack product is less than $5, participants will be able to keep the change from the purchase. If for the selected binding choice scenario, the participant selected "no purchase", the participant will be able to keep the $5. An additional $10.00 will be given to participants in the form of gift cards / vouchers from Stop & Shop, Daily Table or the Big Y, depending on where testing takes place. Vouchers / gift cards will be handed to the participant by a research team member once the survey has been completed.

Participants will be made aware of the compensation amount before beginning the survey. After completing the survey, they will be provided the chosen study product and remaining monetary compensation. Participants will only receive compensation if they complete the experimental portion of the survey.

12.0 Economic Burden:

There is no foreseen economic burden that the participants may bear from participating in this survey. Participants will be given $5 at the beginning of the study so that they don't have an economic burden. All snack products in the choice experiment study are less than $5. The funding for purchase of food products come from the grant funding this study. iPads will be provided to the subjects to complete the survey. Individuals cannot take the survey on their own devices.

Phase 2 – Full Collection

The compensation is the same as in pretesting.

13.0 Recording with Audio, Video, or Photographs

Audio, video and photographic recording will not be used in this research.

14.0 Potential Benefits to Participants:

There are no direct benefits to individual participants. Subjects are informed that the results from the study will inform policy-makers on views related to food and food labeling, which may ultimately benefit them as consumers, although this would be indirect.

15.0 Risks to Participants:

Participants may be inconvenienced by spending unbudgeted time on their shopping trips. The magnitude of this inconvenience is minor. We do not anticipate any foreseeable risks, discomforts, or hazards related to participants' participation in the research. Data collected from pre-testing will only be used for survey improvement and will not be retained for further analysis.

We further do not anticipate any harm to society or non-participants.

16.0 Withdrawal of Participants:

Participants may withdraw of their own free will at any time. We do not anticipate any reason for researchers to remove participants without their consent; if there are concerns about data quality, this will be addressed during the survey improvement process. If a participant chooses to terminate their participation in the study before completion of the survey, their data will not be collected.

17.0 Data Management and Confidentiality:

The research team members will have access to survey responses, but no members will have access to identifying survey response information, as such information will not be collected. No personally identifiable data will be collected, and no data will be retained for further analysis beyond the improvement of the survey for Phase 2. At the time of administration, survey responses will be collected via Qualtrics on an electronic tablet. After survey completion, responses obtained from participants for each question will be stored within the password-protected Tufts Qualtrics site. Data reports will be exported as CSV. files, and stored within a private, Tufts-affiliated Box drive (a secure, cloud-based file storage platform), available only to research team members. No survey data will be stored on the local device. Data will be available to Dr. Cash and other research team members until the team improves the survey instrument, which might take up to 6 months. After this time data will not be retained anymore.

<u>Phase 2 – Full Collection</u>

The management of data and confidentiality are the same as in pretesting. However, data will be available to Dr. Cash and other research team members for up to 2 years. After this time data will not be retained anymore.

18.0 Provisions to Protect the Privacy and Confidentiality of Participants and the Research Data:

Participation in the survey is entirely voluntary. Participants are reminded that they may leave blank any questions they do not wish to answer. Questions do not contain information of a sensitive or personal nature. Participants may skip individual questions, but a complete response will require a response to the choice experiment task questions. Participants will interact with the Tufts team members facilitating the survey's implementation, but no personal information will be provided.

19.0 Provisions to Monitor the Data to Ensure the Safety of Subjects:

No personally-identifying information will be exported from Qualtrics. Data reports will be stored within Box, a private, secure, cloud-based file storage platform. These reports will only be available to the research team.

<u>Phase 2 – Full Collection</u>

The way to monitor the data to ensure the safety of subjects is the same as in pretesting.

20.0 Compensation for Research-Related Injury:

NA

21.0 Data Sharing and Specimen Banking:

No personally identifiable data will be collected, and no data will be retained for analysis. Specimens will not be collected.

22.0 International Research:

NA

23.0 Multiple sites:

NA

24.0 Reliance Agreements/Single IRB:

NA

25.0 Qualifications to Conduct Research and Resources Available:

Dr. Sean Cash is the PI of this study and has extensive experience with survey design, statistical analysis, and food choice studies. Dr. Katherine Fuller, a postdoctoral researcher, has studied and co-authored several papers on consumer preferences, motivations, and willingness to pay. Dr. Shijun Gao is a Post-Doctoral Researcher at the Friedman School of Nutrition Science and Policy at Tufts University. Dr. Gao's research interests include social network analysis, and consumer food choice, especially the impact of food labeling. Caroline Andrews a master's candidate at Tufts University's Friedman School and has a background in food science and professional experience in food product development, including plant-based meat alternatives. Julia Reedy is a Researcher and Senior Research Coordinator in the Mozaffarian Research Lab at Tufts University, whose work includes global dietary data harmonization, food label research, and research translation for U.S. nutrition policy. She received her Master of Nutrition Science and Policy from the Friedman School. Olivia Grieco is a graduate student at Tufts University's Friedman School studying Agriculture, Food, and the Environment. She has a background in food systems with interests in regenerative agriculture, alternative proteins, food policy, and law.

Analysis Plan

Statistical Models

We will run the following models to test our hypothesis.
- A binary logistic regression to assess the proportion of participants that choose healthy and unhealthy snack choices. The main predictor variable is the presence of an FDA Healthy, Healthy, or Food Compass Score. Other predictor variables to analyze are generalized trust, personal trust, institutional trust and trust in the government, knowledge, physical activity levels, diet, and sociodemographics.

We will run the same model for each treatment to test, for example, hypothesis H2.

- We plan to run a multinomial logit model (MNL), random parameter model (RPL), random parameter model with EC (RPL-EC), and latent class analysis (LCA) to investigate participants' willingness to pay on each treatment. We will use the presence of a healthy label or food compass score, and price as the main attributes of the utility function for the MNL, RPL, RPL-EC and LCA. However, for the LCA we will use generalized trust, personal trust, and trust in the government (according to hypothesis H2) as consumers' characteristics for the segment membership function.

Transformations

We will use simple coding for the following categorical variables:

- Income
- Education
- Race

The variable "Diet" will be a dummy variable that takes the value of 0 if participants responded no / not applicable, and the value of 1 if any of the other options is selected.

Inference Criteria

We will use the standard $p<.05$ criteria for the models to determine if the models suggest that the results are significantly different from those expected in the null hypothesis were correct. We will use the Hosmer-Lemeshow test to assess how well the model predicts the dependent variable. In addition, we will use AIC, BIC, log-Likelihood, and p^2 (rho square) to determine the fit of the models. Given the multiple trust indices that we are measuring, we will test for multicollinearity with Variance Inflation Factors (VIF).

We will use statistical tests, such as paired t-tests and ANOVA, to identify the differences between before and after the implementation of the label in the snacks. The same test will be applied to identify differences between treatment results.

Data Exclusion

We will verify that each participant answered each of the twelve choice scenarios for the three treatments. Straightlining: If found that participant will be excluded from the survey. Outliers will be included in the analysis.

Missing Data

If a participant doesn't answer any of the twelve choice scenarios, and / or answer the trust-related questions, that participant will not be included in the analysis.

Exploratory Analysis

If the institutional trust index doesn’t predict the probability of choosing a healthy snack labeled with a Food Compass Score, we will explore the effect of interpersonal trust or generalized trust in the logistic regression.

If the index measuring trust in the government doesn’t predict the probability of choosing an FDA-healthy snack, we will explore the effect of interpersonal trust or institutional trust in the logistic regression.
