# Supplementary material for: Food Compass Score vs FDA Healthy Labeling and Consumer Purchases: A Randomized Clinical Trial
Source: JAMA Netw Open. 2025 Dec 5;8(12):e2546526. doi: 10.1001/jamanetworkopen.2025.46526 (PMC12681036; doi:10.1001/jamanetworkopen.2025.46526)
Supplement: Supplement 3. — Data Sharing Statement [file jamanetwopen-e2546526-s003.pdf]

# Data Sharing Statement

Fan. Effects of Food Compass Score vs FDA Healthy Labeling on Consumer Purchases. *JAMA Netw Open*. Published December 05, 2025. doi:10.1001/jamanetworkopen.2025.46526

**Data**
**Additional Information:** As it is not a clinical study and does not include measurements of a health outcome, this study was pre-registered with the Open Science Foundation (https://doi.org/10.17605/OSF.IO/4JPY7) rather than in clinical trials.gov.
**Data available:** No
